# Long-term CSF responses in adult patients with spinal muscular atrophy type 2 or 3 on treatment with nusinersen

**DOI:** 10.1007/s00415-025-12984-7

**Published:** 2025-03-14

**Authors:** Gina Cebulla, Ling Hai, Uwe Warnken, Cansu Güngör, Dirk C. Hoffmann, Mirjam Korporal-Kuhnke, Brigitte Wildemann, Wolfgang Wick, Tobias Kessler, Markus Weiler

**Affiliations:** 1 Clinical Cooperation Unit Neurooncology, German Cancer Consortium (DKTK), German Cancer Research Center (DKFZ), Heidelberg, Germany; 2 Department of Neurology, Neurology and Neurooncology Program, National Center for Tumor Diseases, Heidelberg University Hospital, Im Neuenheimer Feld 400, 69120 Heidelberg, Germany; 3 Department of Neurology, Heidelberg University Hospital, Im Neuenheimer Feld 400, 69120 Heidelberg, Germany

**Keywords:** Antisense oligonucleotide (ASO), Cerebrospinal fluid (CSF), Mass spectrometry (MS), Nusinersen, Proteomics, Spinal muscular atrophy (SMA)

## Abstract

**Background:**

5q-associated spinal muscular atrophy (SMA) is a monogenic disease causing progressive alpha motor neuron degeneration, muscle atrophy, and weakness. Intrathecal therapy with the antisense oligonucleotide nusinersen modifies the disease course. However, biomarkers for understanding underlying molecular pathomechanisms and monitoring therapy are not yet known.

**Methods:**

A total of 130 cerebrospinal fluid (CSF) samples from 24 adult patients with SMA type 2 or 3 were collected over 3.5 years, and CSF proteome was analyzed using mass spectrometry (MS). By applying two complementary MS protein quantification methods, label-free quantification (LFQ) and tandem mass tag (TMT) isotopic labeling, specific protein patterns reflecting changes in the CSF in response to nusinersen therapy were identified. These results were combined with cellular and metabolic profiles.

**Results:**

Nusinersen therapy led to a median motor function improvement of 2.2 Hammersmith Functional Motor Scale-Expanded points after 10 months and 2.6 points after 34 months. CSF macrophages increased in number and showed an altered morphology. Albumin quotient (qAlb), glucose, and lactate concentrations were inversely correlated with clinical improvement. MS analysis of CSF identified 1,674 (TMT) and 441 (LFQ) proteins. Protein profiles reflected reduced inhibition of “nervous system development” and “axogenesis” pathways under therapy. In addition, clinical improvement was associated with upregulation of the interacting proteins α-dystroglycan and beta-1,4-glucuronyltransferase 1, reduction of complement factors, negative correlation in immunoglobulin- and B cell-related pathways, and reduction of cellular mediators such as lymphocytes.

**Conclusion:**

The present multi-proteomic analysis contributes to the understanding of the molecular mechanisms underlying nusinersen’s therapeutic effects and offers potential biomarkers for monitoring treatment response in SMA.

**Supplementary Information:**

The online version contains supplementary material available at 10.1007/s00415-025-12984-7.

## Introduction

5q-associated spinal muscular atrophy (SMA) is a rare autosomal recessive neuromuscular disorder characterized by the progressive loss of motor neurons in the spinal cord, resulting in muscle weakness and atrophy [1]. Disease symptoms arise from a deficiency of functional survival motor neuron (SMN) protein, caused by a homozygous deletion or, less commonly, loss-of-function mutations in the *SMN1* gene, resulting in reduced SMN protein levels [1]. The paralogous *SMN2* gene partially compensates, but most of its transcripts lack exon 7 due to alternative splicing, leading to non-functional SMN protein [1, 2].

Nusinersen, an antisense oligonucleotide (ASO), is administered via periodic intrathecal injection. It modifies the splicing of the *SMN2* gene by promoting the inclusion of exon 7 in the *SMN2* mRNA transcript and thereby increasing the production of functional full-length SMN protein [3, 4]. Studies have demonstrated the efficacy of nusinersen in pediatric and adult patients [5–9]. Nevertheless, significant inter-patient variability in therapeutic response necessitates the development of reliable biomarkers and robust monitoring tools. Despite this need, the identification of such biomarkers remains challenging.

This study focuses on cerebrospinal fluid (CSF) analysis, as spinal cord biopsy material for molecular analysis of anterior horn cells is unavailable. CSF, due to its direct contact, likely serves as the most informative surrogate biomaterial, despite SMN being an intracellular protein. Early studies focused on targeted proteomics in CSF, proposing biomarkers related to disease activity, but comprehensive molecular changes were not fully elucidated [10–16]. Recent non-targeted proteomic investigations revealed disrupted blood–brain barrier integrity, identified key markers in the insulin-growth factor pathway, and proposed individual disparate CSF proteins predictive of clinical improvement [17–21], emphasizing the need for a broader understanding of nusinersen’s impact on molecular dynamics in SMA patients.

The present work includes a cohort of 24 individuals over an observation period of up to 3.5 years with seven observational time points. In addition, we used an enhanced methodology by integrating two complementary mass spectrometry (MS) protein quantification methods for CSF analysis. By incorporating tandem mass tag (TMT) labeling and fractionation into the MS workflow, our study delved deeper into the proteome, enabling the detection of alterations in less abundant proteins that may play crucial roles.

## Methods

### Standard protocol approvals, registration, and patient consents

This prospective, monocentric study was approved by the institutional ethics board of the University of Heidelberg (S-554/2018) and adheres to the ethical principles outlined in the Declaration of Helsinki (World Medical Association) [22]. Written informed consent was obtained from all participants included in the study.

### Patient enrollment and clinical assessments

A total of 24 patients (13 male, 11 female) were consecutively enrolled at the Department of Neurology at Heidelberg University Hospital between December 2017 and October 2020. The inclusion criteria comprised a clinical and molecular diagnosis confirming 5q-associated SMA type 2 or 3, and a minimum age of 18 years. Ten of these patients have been included in a previous study by our group [17]. Classification of SMA 2 or 3 was determined clinically, considering the age of symptom onset and attainment of motor milestones. The clinical severity of symptoms was evaluated by experienced physiotherapists specialized in SMA (Heidi Rochau-Trumpp and Guido Stocker, each with over 20 years of professional experience and more than 8 years dedicated to SMA patients). The evaluation of motor functions was assessed by the Hammersmith Functional Motor Scale-Expanded (HFMSE), a validated rating scale designed for the assessment of motor functions in patients with SMA [23–25].

### Sampling

**Fig. 1:**
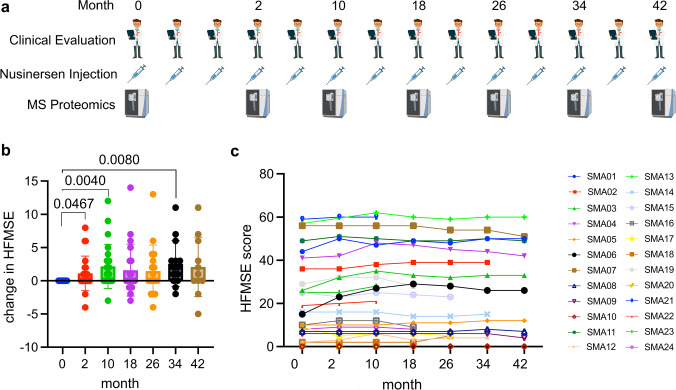
Study design and evaluation of nusinersen treatment effects. **a** Timepoints of nusinersen applications, clinical evaluation and MS analysis. **b** Clinical change of HFMSE compared to baseline. Only significant *P* values are plotted. **c** Time course of individual HFMSE scores

CSF was collected and clinical scores were assessed at up to 7 timepoints per patient, with the initial collection occurring prior to the first administration of nusinersen and subsequent time points at 2 months (4th nusinersen administration), 10 months (6th nusinersen administration), 18 months (8th nusinersen administration), 24 months (10th nusinersen administration), 34 months (12th nusinersen administration), and 42 months (14th nusinersen administration). The chronological layout is depicted in Fig. 1a. Data were obtained for all 7 time points for 11 patients, from 3 patients for 6, 5, and 4 time points, respectively, and from 4 patients for the initial 3 time points. Patients with data available for fewer than three time points were excluded. Lumbar puncture procedures and intrathecal administrations of nusinersen were conducted between 8 a.m. and 11 a.m. For patients presenting anatomical challenges for conventional lumbar punctures, the intrathecal administration of nusinersen was facilitated using fluoroscopy or computed tomography guidance. The CSF collection was followed by intrathecal administration of nusinersen (12 mg dissolved in a sterile, preservative-free solution). Following the transfer of nusinersen from its single-dose glass vial into a single-use plastic syringe under sterile conditions, each injection was administered within 1–3 h. The antisense oligonucleotide was sourced directly from the manufacturer (Biogen) through the pharmacy at Heidelberg University Hospital.

### Routine CSF analysis

Following the lumbar puncture, CSF was collected in polypropylene tubes (Falcon 15 ml Conical Centrifuge Tubes; Thermo Fisher Scientific). Within the initial hour post-collection, routine CSF parameters, encompassing cell count, cytology, glucose, lactate, total protein, albumin and CSF/serum quotients of albumin, were assessed. Manual cell counting transpired using a Fuchs-Rosenthal counting chamber (Brand). A centrifugation step of 5–10 ml CSF for 5 min at 700 g in a Hettich Rotina 35 (Hettich) was executed to concentrate the cells. The resultant supernatant was preserved, frozen, and stored at − 80 °C until readiness for preparation in anticipation of proteomic analysis.

### Cytology

The cellular sediment underwent resuspension in 0.2 ml of cell culture medium, followed by the meticulous preparation of cytospins using a Shandon Cytospin 3 (Thermo Shandon Limited) for cytological analysis. These cells were delicately air-dried and subsequently subjected to staining using the panoptic Pappenheim technique. This involved sequential May–Grünwald (Merck) and Giemsa (Merck) staining procedures. The distinctive cell populations were then counted, with the total amount being 100 cells per slide. The percentage of each cell type was then multiplied by the total cell count per 100 µl to calculate the absolute number of each cell type per 100 µl CSF per patient.

### Mass spectrometry sample processing

130 fresh frozen CSF samples were prepared for label free as well as TMT protein MS quantification.

Acetone precipitation: The amount of protein utilized per sample varied based on the number of time points investigated per patient, ranging from 400 µg for three time points to 200 µg for seven time points. Four times the calculated volume of acetone, comprising 80% acetone and 20% NaCl, was added to the protein sample. The mixture was thoroughly mixed to achieve homogeneity. The sample underwent centrifugation at 10,000 rotations per minute for a duration of 5 min, followed by the removal of the supernatant to preserve a protein pellet. Following acetone precipitation, the sample was briefly subjected to drying in a SpeedVac until complete dryness of all samples was achieved.

Protein digestion: The protein pellets were lysed in 200 µl of a lysis buffer containing 8 M urea, 50 mM pH 8.5 tetraethylammonium bromide, 1 mM NaCl, 1% Benzonase (Sigma), one Complete Tablets Mini EDTA-free EASY pack protease inhibitor tablet (Complete Tablets Mini, EDTA-free EASY pack), and PhosSTOP protease inhibitor buffer (Sigma-Aldrich) per 10 mL of urea buffer. Protein reduction and alkylation were carried out at 27 °C using 10 mM dithiothreitol, followed by treatment with 30 mM iodoacetamide for 1 h at room temperature. Protein digestion was performed overnight at 37 °C using Trypsin/Lys-C (Promega) at a 1:50 enzyme-to-protein ratio. To terminate the reaction, 10% formic acid (FA) was added to achieve a final concentration of 2% FA. A 50 µl aliquot was saved for subsequent single-shot label-free quantification (LFQ) analysis. The rest of the samples were purified according to the protocol for Pierce Peptide Desalting Columns (Thermo Fisher Scientific).

### TMT labeling

The peptides each sample were labeled with TMT 6plex isobaric tags (Thermo Fisher Scientific) at room temperature for 1 h, following the manufacturers protocol, with sample-to-TMT reagent ratios modified as recommended in the work by Zecha et al. [26]. In summary, for every sample we employed a mass of TMT label reagent that was twice the mass of the peptides. In the case of a seventh time point sample in a patient series, the additional peptide samples were labeled with the TMT11-131C Label Reagent (Thermo Fisher Scientific). Due to this, the MS settings and the downstream data processing were handled according to 11plex labeling experiments.

### Sample fractionation

Fractionation of the TMT-labeled peptides was performed using the Pierce™ High pH Reversed-phase Peptide Fractionation Kit (Thermo Fisher Scientific) according to the manufacturer’s protocol. Briefly, the samples were dissolved in 300 µL of 0.1% trifluoroacetic acid (TFA) and loaded onto a spin column. Eight fractions were eluted with increasing gradients of acetonitrile (MeCN) by centrifugation. In addition, the initial “flow-through” fraction was collected for analysis. Collected fractions were dried via vacuum centrifugation and re-suspended in 2.5% hexafluoro-2-propanol and 0.1% TFA for LC–MS analysis.

### NanoLC–MS/MS proteome analysis

Peptide samples prepared for global proteome and TMT isotope-labeled analysis were separated and analyzed by nanoflow LC–MS/MS using a Dionex 3000 nanoUHPLC in line with an Orbitrap Exploris 480 mass spectrometer (Thermo Fisher Scientific). A trapping column Acclaim PepMap300 C18, 5 μm, 300 Å (Thermo Fisher Scientific) was used for peptide loading and washing. Peptides were separated on a nanoEase, 1.7 μm, 300 Å, 75 μm × 200 mm analytical column (Waters) at a flow rate of 300 nl/min. For LFQ proteome analyses 30 min and for TMT analysis 150 min, three-step linear gradients were applied for chromatography. Starting with a constant flow of solvent A (99.9% water, 0.1% FA) for 2 min, the gradient steps were set to 2–12% of solvent B (99.9% MeCN, 0.1% FA) in 3 min, 12−22% in 10 min and 22–35% in 5 min and finally by a 2 min hold at 76% solvent B. For reconditioning, a 8-min washing and equilibration step with solvent A was applied. The gradient for 150 min TMT-labeled peptide separations was adjusted as: 2–4% solvent B in 1 min, 4–30% in 132 min and 30–80% in 2 min at a final hold at 1 min followed by a 10 min washing and an equilibration step with solvent A. The spray voltage was 2.2 kV for nanoESI ionization and the ion transfer tube temperature was set to 275 °C.

The Orbitrap was operated in the data-dependent (DDA) mode for the 30 min LFQ runs as well for the 150 min isotype peptide-labeled TMT runs. For 30 min DDA runs, full-scan MS spectra (m/z 300–1500) were acquired with a maximum injection time of 45 ms at 60,000 resolution in the MS1 mode. The automatic gain control (AGC) target value was set to 300% and the isolation window was set to 1.2 m/z. The normalized MS collision energy was set to 26. MS/MS scans cycles were triggered for 2 s. A maximum injection time of 50 ms at 15,000 resolution was set for high-resolution MSMS spectra. Dynamic exclusion was set to 60 s. Undetermined charge states and single charged signals were excluded from fragmentation.

For 150 min TMT runs, full-scan MS spectra (m/z 300–1400) were acquired with a maximum injection time of 45 ms at 120,000 resolution. The AGC target value was set to 100% and the isolation window was 0.7 m/z. The normalized MS collision energy was set to 38. MS/MS scans cycles were triggered for 2 s with a maximum injection time of 50 ms at 45,000 resolution.

Protein identifications and quantifications were performed with the MaxQuant v.2.1.3.0 (RRID:SCR_014485) software package including Perseus v.2.0.6.0 (RRID:SCR_015753) for statistical analysis. Proteins were identified applying the UniProt database UP000000589 (Homo sapiens; 01, 2020; 20,378 sequences, RRID:SCR_002380). Carbamidomethylation of cysteines was set as fixed modification. Oxidation of methionine, N-terminal acetylation, glutamine and asparagine deamidation were set as variable modifications. The ‘match-between-runs’ function and LFQ option were enabled for 30 min DDA raw data. For TMT labeled peptide analysis, the 11plex MS2 reporter ion identification and quantification processing strategy embedded in MaxQuant was applied to the raw data. DDA and TMT sample false discovery rate (FDR) cutoffs were 0.01 on the protein level and peptide level. To exclude false positive protein identifications, Perseus protein identification filtering was performed by ‘only identified by site’ and identified by ‘reverse’ protein database. Utilizing Perseus, unoccupied TMT reporter ion channels were excluded for further quantification analysis.

### Proteomics data preprocessing

Data processing and statistical analysis were performed using R (v.4.1.0, RRID: SCR_001905). In the TMT data, two samples with lower total intensity (< 225) than others were removed. In both the TMT and LFQ data, proteins detected in at least two samples within the same patient, as well as those detected in at least two patients, were kept for further analysis.

The intensities of proteins were normalized using the variance stabilizing transformation method within the DEP package (v.1.14.0, RRID:SCR_023090). Since the missing values of proteins were missing at random, they were imputed using the k-nearest neighbor approach within the DEP package.

### Sample correlation analysis

The normalized and imputed intensities of proteins were adjusted for heterogeneity between patients using the removeBatchEffect function of the limma (v.3.36.5, RRID: SCR_010943) package. Subsequently, the corrected values were scaled into z scores, and then Pearson correlation between samples was calculated. The Pearson correlation coefficients were visualized in heatmaps using the ComplexHeatmap package (v.2.5.4, RRID: SCR_017270), with hierarchical clustering on both rows and columns.

### Differentially expressed protein analysis

The normalized and imputed intensities of proteins were utilized for the analysis of differentially expressed proteins (DEPs) using the DEP package (v.1.14.0, RRID:SCR_023090). Pairwise comparisons between time points within all patients were conducted using the test_diff function. For adjusting heterogeneity between patients in the DEPA, a custom design formula (~ 0 + time point + patient) was employed. *P* values were adjusted for multiple testing using the FDR method. Proteins with an FDR < 0.05 and log2 fold changes > 0.5 between time points were identified as DEPs. Visualization was conducted with ggplot2 package as well as GraphPad Prism (Version 10.1.1).

### Statistical analysis

All remaining longitudinal analyses, except protein analyses, were conducted using a mixed-effects analysis with Geisser–Greenhouse correction and Fisher’s LSD post hoc test, with no correction for multiple testing. Number of data points included were: *n* = 24 patients (0, 2, 10 months), *n* = 20 patients (18 months), *n* = 17 patients (26 months), *n* = 14 patients (34 months), and *n* = 11 patients (42 months).

For scatter plot correlation analyses of changes in different features with changes in protein intensities, 130 data points from 24 patients repeatedly assessed over 7 time points were used. A two-sided Pearson correlation test was performed, and exact *P* values are shown in the figures.

Boxes in box plots represent the 25–75th percentile, the middle line represents the median, whiskers represent the minimum to maximum (excluding outliers), and individually plotted data points indicate outliers. ANOVA was performed, and exact *P* values are shown in the figures.

Visualization was conducted with ggplot2 package as well as GraphPad Prism (Version 10.1.1, RRID:SCR_002798).

### String database protein interaction analysis

Protein–protein interaction networks were conducted using the String database (STRING: functional protein association networks (string-db.org); RRID:SCR_005223). Default settings were used, except the following ones: ‘Evidence’ as meaning of network edges, ‘High confidence (0.700)’ as minimum required interaction score.

## Results

### Study design and patient characteristics

Among the 24 patients enrolled, 3 had SMA type 2, 10 had type 3a, and 11 had type 3b. Twenty patients exhibited homozygous deletions of exons 7/8 within the *SMN1* gene, while the remaining four patients had heterozygous deletions coupled with point mutations. The mean *SMN2* copy number was 3 (± 0.2, range 2–4). Average age of the patient cohort was 33.1 years (± 2.3, age range 18–50 years), and the average duration of symptoms was 26.4 years (± 2.1, range 6–46 years) (Supplementary Table 1). Nusinersen administration and clinical evaluation was performed four times within the first 9 weeks, then on a 4-month basis. MS analysis was conducted at baseline, after 2 months, and then every 8 months (Fig. 1a). Motor abilities were regularly monitored by determining the HFMSE score at each clinical assessment. The mean baseline HFMSE score prior to the initial nusinersen dose was 23.0 (± 3.9, range 0–59 points). Nusinersen administration resulted in an average improvement of 1.1 (± 0.5) points at month 2 (24 patients included), 2.2 (± 0.7) points at month 10 (24 patients), 1.6 points (± 0.9) at month 18 (20 patients), 1.4 (± 1.0) at month 24 (17 patients), 2.6 (± 0.9) at month 34 (14 patients) and 2.1 (± 1.4) at month 42 (11 patients) (Fig. 1b). Overall, there was a high inter-patient variance (Fig. 1c).

### *SMN2* copy numbers and pre-treatment physical functionality do not influence clinical response to nusinersen

We then examined whether the degree of clinical alteration with nusinersen therapy could be attributed to genetic or clinical characteristics. HFMSE score alterations after 10 months, the latest available time point for all included patients, and after 34 months, to account for any later response differences, were analyzed (Supplementary Fig. 1).

There was no association between *SMN2* copy numbers and the clinical response to nusinersen (Supplementary Fig. 1a) in our cohort. There was also no distinction in the treatment response among SMA patients based on the SMA type, i.e., 2, 3a, 3b (Supplementary Fig. 1b), or functional classifications differentiating non-sitters, sitters, and walkers (Supplementary Fig. 1c). We further investigated a potential correlation of the clinical alterations after 10 and 34 months, respectively, with the initial HFMSE scores (Supplementary Fig. 2a), the age of treatment onset (Supplementary Fig. 2b), and the duration of clinical symptoms (Supplementary Fig. 2c) but did not find any support for an association in our cohort.

### CSF qAlb, lactate, and glucose levels show a negative correlation with motor improvement

**Fig. 2:**
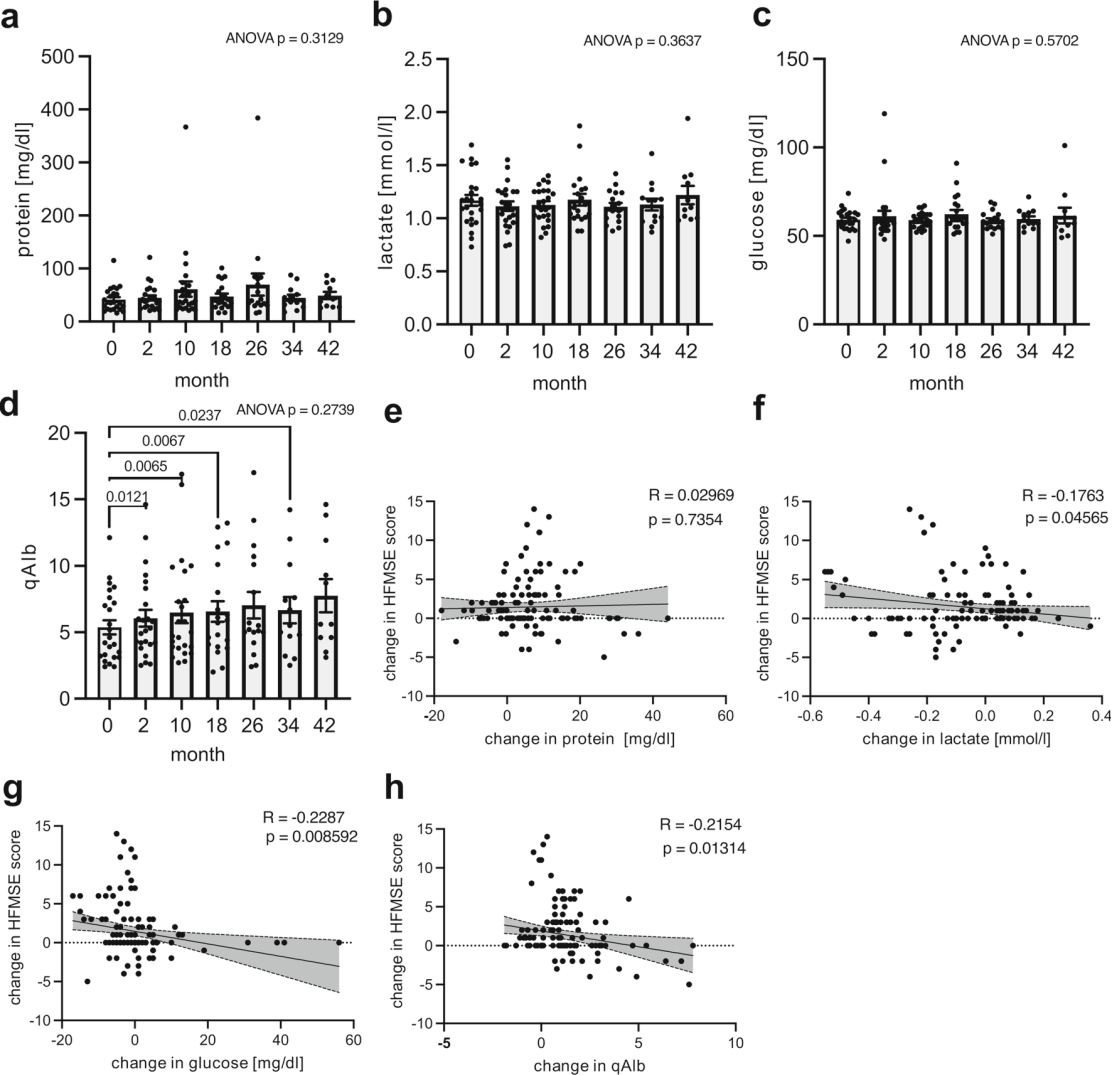
CSF metabolite levels and correlated features. **a–d** Longitudinal evolution of metabolite profiles under nusinersen treatment. **a** Protein concentration. **b** Lactate concentration. **c** Glucose concentration. **d** qAlb. **a–d** *n* = 24 patients (0, 2, 10 month) vs *n* = 20 patients (18 month) vs *n* = 17 patients (26 month) vs *n* = 14 patients (34 month) vs *n* = 11 patients (42 month). Mixed-effects analysis with Geisser–Greenhouse correction and Fisher’s LSD post hoc test. No correction for multiple testing was performed. Only significant *P* values are plotted. **e–h** Clinical improvement measured by HFMSE correlated with change in CSF basic parameters under nusinersen treatment. **e** Change in protein concentration. **f** Change in lactate concentration. **g** Change in glucose concentration. **h** Change in qAlb. **e–h** *n* = 130 data points from *n* = 24 patients repeatedly assessed over *n* = 7 time points. Two-sided Pearson correlation test. Exact *P* values are shown in the figure

We examined whether CSF parameters exhibited alterations throughout the course of the treatment. Within our cohort, no discernible variations in protein, glucose, or lactate levels were observed (Fig. 2a–c). However, the albumin quotient (CSF/serum) (qAlb), the latter being a marker for blood influx to the CSF [27], was upregulated at individual timepoints within the initial months of treatment (Fig. 2d).

Next, we assessed whether changes in basic CSF parameters corresponded with clinical improvements. Of note, we used a continuous scale to quantify clinical changes of motor function rather than relying on the conventional three-point HFMSE cutoff. This approach was chosen to achieve a better resolution and, consequently, a more precise assessment of the development of clinical status.

We identified a significant negative correlation between HFMSE improvement and alterations in qAlb, lactate and glucose, whereas the overall protein amount remained unaffected (Fig. 2e–h). However, the substantial inter-patient variabilities resulting in weak overall correlations might limit the potential of these molecular alterations as reliable biomarkers for individual patients. Thus, we strived our interest towards alternative biomarkers to better predict treatment efficacy and monitor therapy.

### Nusinersen-induced morphological alterations in macrophages and reduced lymphocyte counts in clinical responders

**Fig. 3:**
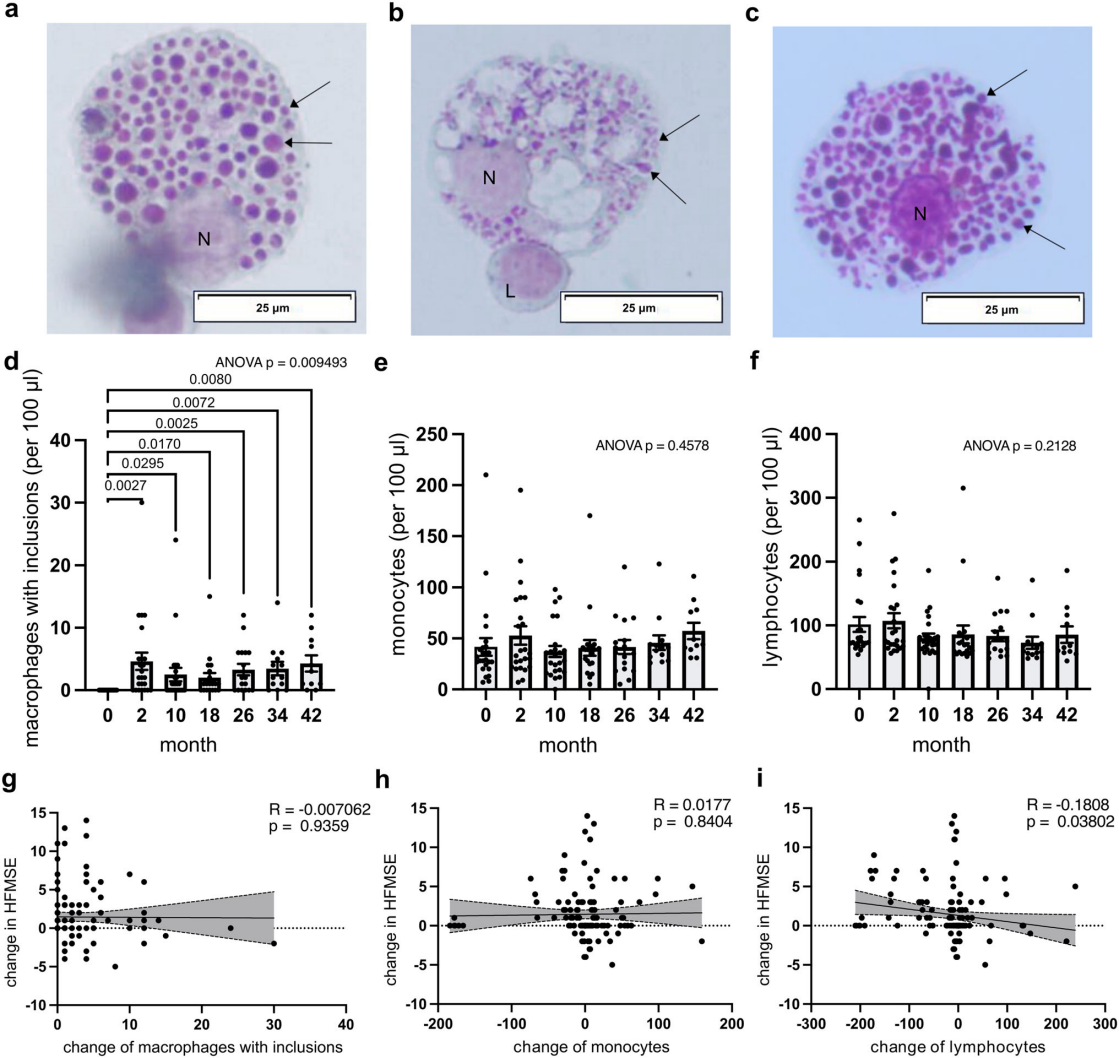
CSF cytology and cell counts. **a–c** Macrophages with characteristic inclusions **a**, **b** after 2 months of nusinersen treatment: **a** Patient SMA04, **b** Patient SMA11, and **c** after 34 months of treatment in patient SMA11. **a–c** Arrow: characteristic inclusion, N: nucleus, L: lymphocyte. **d–f** Longitudinal evolution of cellular profiles under nusinersen treatment: **d** macrophages, **e** monocytes, **f** lymphocytes. **d–f** *n* = 24 patients (0, 2, 10 month) vs *n* = 20 patients (18 month) vs *n* = 17 patients (26 month) vs *n* = 14 patients (34 month) vs *n* = 11 patients (42 month). Mixed-effects analysis with Geisser–Greenhouse correction and Fisher’s LSD post hoc test. No correction for multiple testing was performed. Only significant *P* values are plotted. **g–i** Clinical alterations measured by HFMSE correlated with change in CSF parameters under nusinersen treatment. *n* = 130 data points from *n* = 24 patients repeatedly assessed over maximum of *n* = 7 time points. Two-sided Pearson correlation test. Exact *P* values are shown in the figure. **g** Change in macrophages (per 100 ml). **h** Change in monocytes (per 100 ml). **i** Change in lymphocytes (per 100 ml)

In CSF specimens from patients treated with nusinersen, we observed macrophages exhibiting a morphology with intracellular inclusions (Fig. 3a–c). This cell type was absent in patients before nusinersen treatment was started. This finding had been described in previous studies investigating SMA patients treated with nusinersen [28–30]. The immediate and continuous presence of these inclusion-bearing macrophages was seen from the 2-month time point on (Fig. 3d), which was the first evaluated time point after treatment initiation, suggesting increased macrophage counts as a response marker for nusinersen or possibly as a mechanism of resistance. However, a concomitant increase in monocyte or lymphocyte counts within the CSF over the 3.5-year treatment period was not observed (Fig. 3e, f).

Furthermore, we correlated the changes in CSF cytology throughout nusinersen treatment with corresponding clinical alterations assessed by the HFMSE. Surprisingly, no discernible correlation emerged between the presence of macrophages and clinical response (Fig. 3g), thus rendering macrophage levels as a response indicator but not a suitable monitoring biomarker. A similar trend was observed in monocytes (Fig. 3h). Conversely, an intriguing negative correlation was seen between the changes in lymphocyte counts and HFMSE alterations (Fig. 3i), indicating that lymphocyte-mediated inflammation was diminished in patients exhibiting motor improvement. However, as with the CSF basic parameters, lymphocyte correlations displayed substantial inter-patient variability, limiting their reliability as a single biomarker for individual patients.

### A robust dual proteomic workflow for the analysis of CSF in SMA

**Fig. 4:**
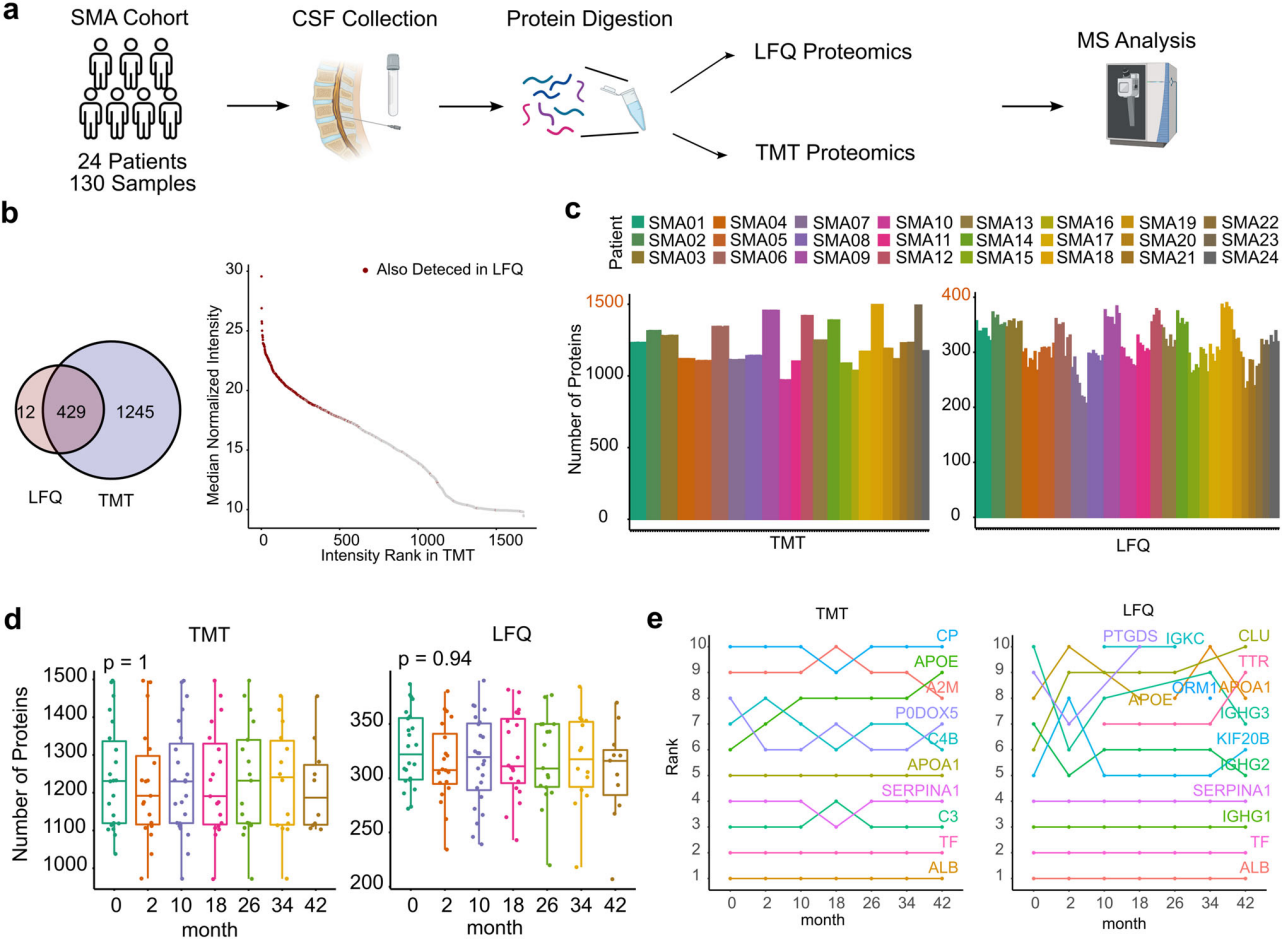
Workflow and comparison of TMT and LFQ proteomics. **a** Overview of the workflow. **b** Overlap of identified proteins of both proteomic methods. **c** Rank intensity of TMT proteins with colored overlap of proteins identified in LFQ proteomics. **d** Number of identified proteins across all samples from all patients in TMT and LFQ. **e** Number of identified proteins per timepoint in TMT and LFQ. **f** Rank of top ten most abundant proteins with rank changes over time

We next sought to identify protein biomarkers from CSF predictive for longitudinal nusinersen effects. With the intention to combine the distinct advantages of two MS quantification methodologies, a more sensitive TMT label-based approach as well as a more accurate shotgun approach using LFQ values were employed (Fig. 4a). The TMT methodology yielded 1,674 identified proteins over all patients and samples, whereas the LFQ shotgun approach detected 441 proteins. Notably, out of 441 proteins identified in the LFQ approach, 429 were also identified using the TMT approach (Fig. 4b). The shared proteins predominantly belonged to the higher abundance range, whereas the lower abundant proteins were only captured by the TMT-based approach (Fig. 4c). Both methods similarly captured trends in the number of identified proteins between patient individuals (Fig. 4d) and robustly reflected that protein numbers did not alter over time (Fig. 4e). In line, most of the top 10 highest abundant proteins did not change over time relative to each other (Fig. 4f). Albumin was consistently identified the most abundant CSF protein over time (Fig. 4f). Overall, the workflow harnesses a dual strategy, thus providing a robust analytical framework through boosting the confidence in commonly identified targets as well as reducing the method bias and number of potential false negatives.

### Longitudinal SMA proteomes reflect a shifting protein composition and modulation of pathways related to “nervous system development” and “axogenesis” on treatment with nusinersen

**Fig. 5:**
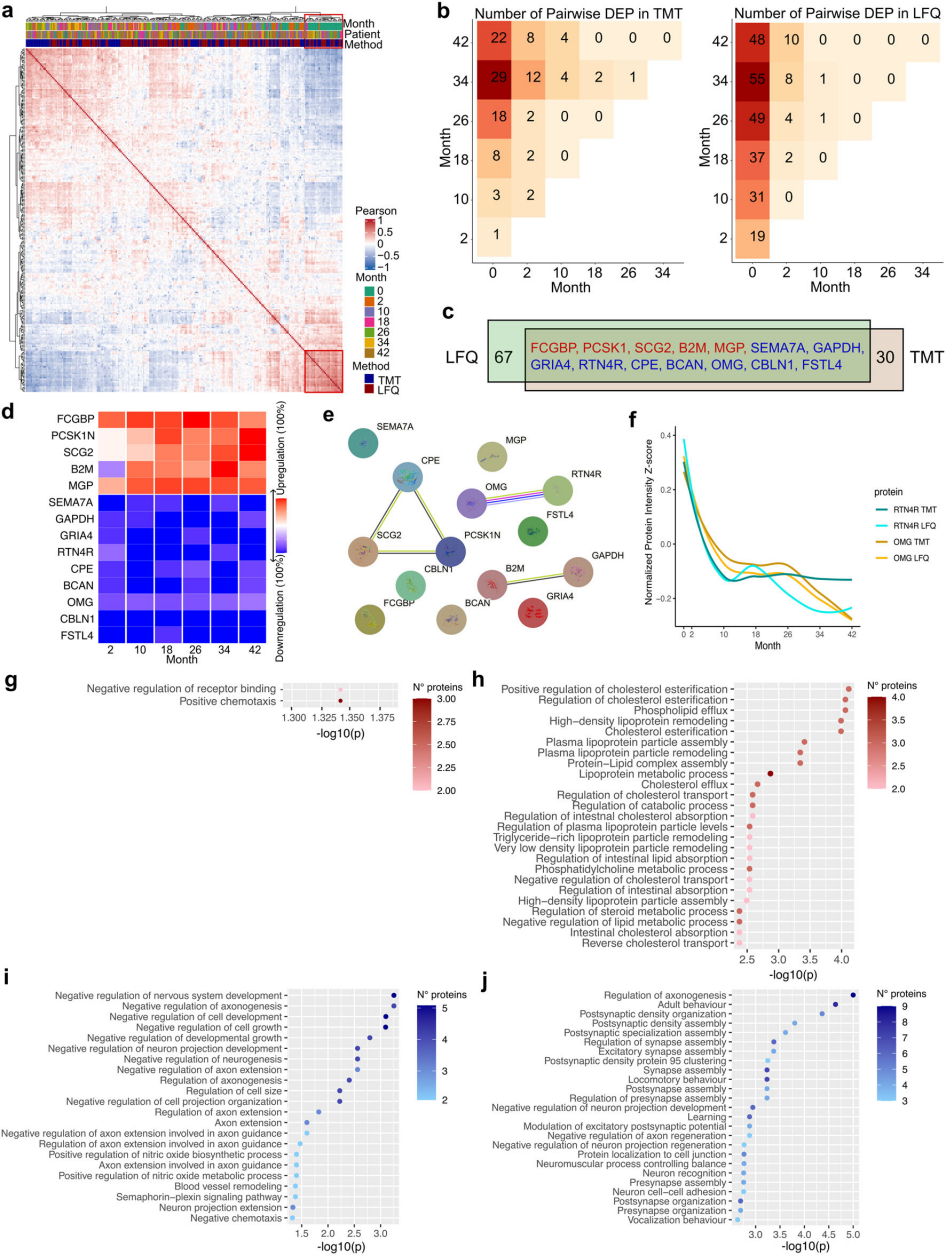
Longitudinal analysis of CSF protein changes under nusinersen treatment. **a** Heatmap of all analyzed samples (*n* = 260) from all patients (*n* = 24) of TMT and LFQ proteomics. **b** Number of pairwise DEPs over all analyzed timepoints (*n* = 7). **c** Overlap of DEPs. **d** Heatmap of the percentage of patients showing regulation in the corresponding direction for all timepoints in all overlapping DEPs (based on the TMT dataset). **e** STRING database analysis of overlapping DEPs. **f** RTN4R and OMG z-Intensity scores evolution under nusinersen treatment. **g–j** GO terms of identified longitudinal DEPs under nusinersen treatment. Specifically GO terms of: **g** upregulated DEPs in TMT proteomics, **h** upregulated DEPs in LFQ proteomics, **i** downregulated DEPs in TMT proteomics, **j** downregulated DEPs in LFQ proteomics

To better understand whether and, if so, when treatment with nusinersen was accompanied by proteomic changes, we performed unsupervised hierarchical clustering of the protein profiles of all samples using both TMT and LFQ approaches. Pre-therapy samples revealed low diversity and strong similarity, whereas samples collected during treatment clustered separately (Fig. 5a), suggesting a general shift of the protein composition. Notably, there were no subclusters enriched for any other timepoint or patient, reflecting an inter-patient heterogeneity of the molecular response. To identify deregulated proteins throughout the course of nusinersen administrations, we conducted DEP analysis for each timepoint in both TMT and LFQ data. Consistently between both methods, the majority of DEPs were identified in comparisons between the pre-treatment and following timepoints (Fig. 5b). This, along with the increased macrophage counts after 2 months of treatment (Fig. 5c), suggests that nusinersen initiates the majority of effects already early after administration. However, the molecular execution of these effects becomes progressively evident over time, achieving statistical significance only after several months. The number and type of DEPs identified with each respective proteomic quantification method were, however, distinct. Forty-four DEPs were identified through the TMT approach and eighty-one DEPs through the LFQ method (Fig. 5c, Supplementary Fig. 3). Five upregulated and nine downregulated proteins exhibited consistent differential expression across both analyses (Fig. 5c). To determine the relevance of these proteins as potential biomarkers, we assessed individual patient responses at each timepoint (Fig. 5d) in each upregulated (Supplementary Fig. 4,) and downregulated (Supplementary Fig. 5) DEP. After 10 months, 12 of the 14 proteins showed regulation in the corresponding direction in at least 85% of patients, with 6 proteins exhibiting consistent regulation across 100% of patients (Fig. 5d, Supplementary Figs. 4, 5). After 34 months, this consistency remained high, with 12 of 14 proteins regulated in at least 85% of patients, and 8 proteins displaying uniform regulation in all patients (Fig. 5d, Supplementary Figs. 4, 5**)**. To explore relevant signaling pathways from potential molecular interactions of these proteins, we leveraged the STRING database. Oligodendrocyte Myelin Glycoprotein (OMG) and Reticulon-4 receptor (RTN4R) emerged as the only functionally validated receptor–ligand pair among the shared proteins (Fig. 5e), both exhibiting joint downregulation over time (Fig. 5f). Previously, the interaction between OMG and RTN4R has been described a key barrier to axon regeneration in the adult CNS after injuries [31]. Of note, OMG showed a downregulation in 85.7% of patients after 10 months of treatment (84.6% after 34 months) while RTN4R showed a downregulation in 100% of patients after 10 months (92.3% after 34 months).

To mine the biologic context of all upregulated and downregulated proteins on a systematic level, we conducted Gene Ontology (GO) term analysis. While the analysis of upregulated proteins in the TMT methodology revealed only two GO terms with low significance (Fig. 5g), the main semantic groups delineated from the LFQ dataset included cholesterol and lipoprotein metabolism. (Fig. 5h).

The examination of downregulated DEPs exhibited a high significance in pathways like “negative regulation of nervous system development”, “negative regulation of neurogenesis”, and “negative regulation of axogenesis” (Fig. 5i). In addition, there was an enrichment of pathways involved in the “negative regulation of cell development” and “negative regulation of cell growth”. These findings align with the observation that patients overall show improvement in their motor skills over the course of nusinersen treatment. Likewise, these nervous system development-related semantic groups were also found in the LFQ dataset (Fig. 5j).

### Clinical improvement with nusinersen is accompanied by an upregulation of the interacting proteins DAG1 and B4GAT1, reduction of certain complement factors, and a less activated immune response

**Fig. 6:**
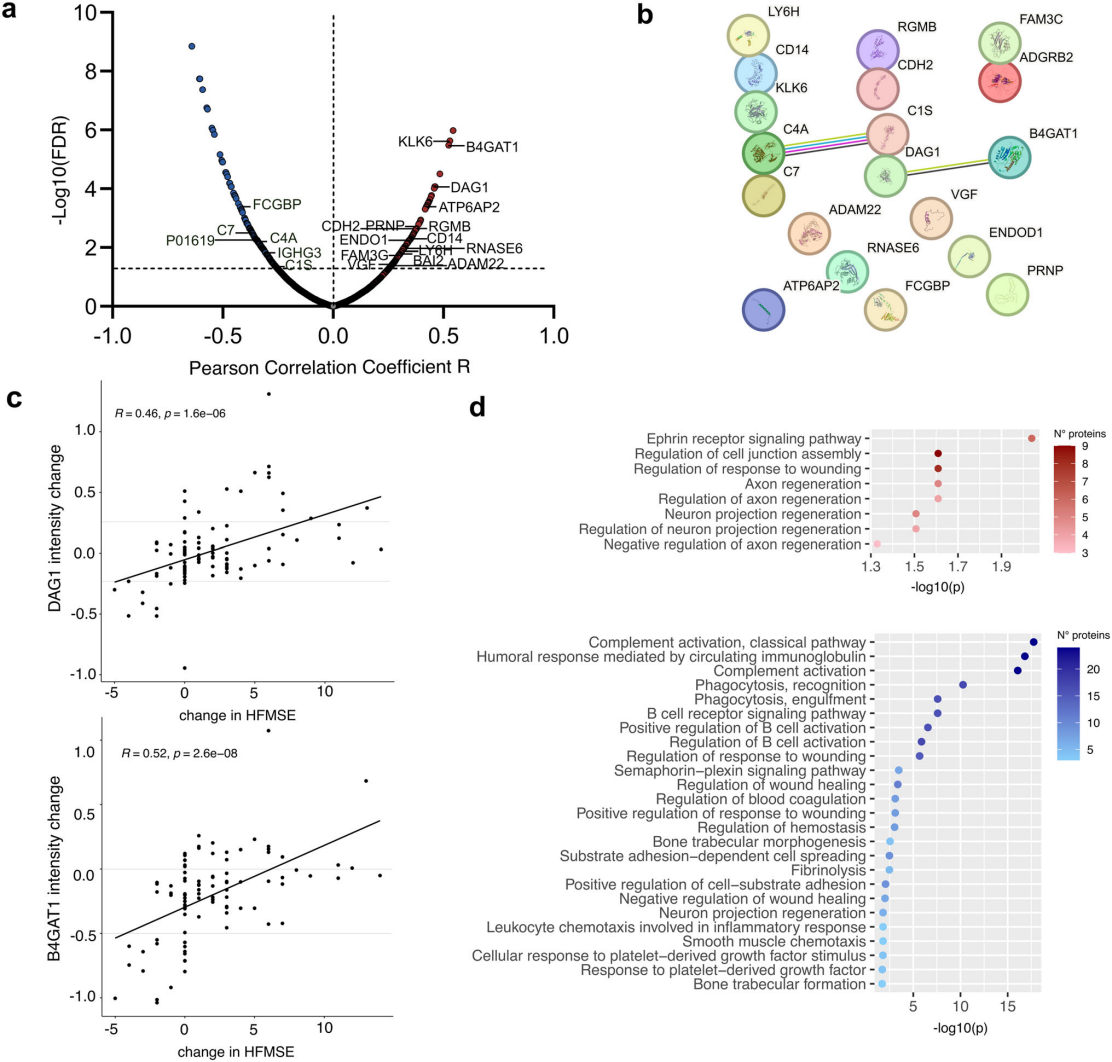
Clinical correlation of CSF protein changes with HFMSE alterations.** a** Volcano plot depicting TMT-identified protein changes correlated with HFMSE alterations; protein changes with significant correlation in LFQ are highlighted.** b** Protein–protein interaction network using proteins exhibiting significant correlation in their changes with HFMSE alterations in both quantification methods.** c** Correlations of HFMSE-based clinical alterations and DAG1 and B4GAT1 protein expression levels (TMT MS analysis). **d** GO term analysis of positively (red) and negatively (blue) correlated protein changes with HFMSE alterations in TMT proteomics

To identify potential monitoring biomarkers for clinical response to nusinersen, individual patients’ protein intensity changes were systematically correlated with changes in HFMSE-measured motor skills. Of those proteins that exhibited significant correlation with clinical alterations, 21 were mutually identified in both proteomic analyses. Of these, 15 displayed positive correlations, while 6 exhibited negative correlations (Fig. 6a). The STRING database was again utilized to search for potential molecular interactions, identifying two significant interactions (Fig. 6b). One involved C4A and C1S, two complement factors, both of which were downregulated in patients demonstrating improved motor skills. The other interaction involved α-dystroglycan (DAG1) and beta-1,4-glucuronyltransferase 1 (B4GAT1), a receptor–ligand pair positively correlated with HFMSE alterations (Fig. 6c). Their interaction is described to be crucial for neuromuscular integrity and contributes to the pathomechanism in muscular dystrophies [32, 33]. Underlining their relevance, their Pearson correlation values ranked among the top three highest of all shared DEPs (Fig. 6a, c). Further analysis revealed that DAG1 and B4GAT1 protein intensities may serve as candidate biomarkers for clinical improvement. Patients with a DAG1 intensity increase of ≥ 0.25 exhibited clinical improvement or stability, whereas a decrease of ≥ 0.25 in intensity was associated with no improvement or worsening (Fig. 6c). Similarly, an increase in B4GAT1 protein intensity was consistently linked to clinical improvement or stability, while a decrease by at least − 0.5 in intensity was observed exclusively in patients with clinical worsening or lack of improvement (Fig. 6c).

Next, systems biology approaches were applied to investigate common biologic functions of proteins whose expression levels correlated with changes in motor skills (Fig. 6d). The “Ephrin receptor signaling pathway” emerged as the most significant GO term derived from positively correlated DEPs. The pathway is known to play a crucial role in governing guidance for motor neuron axons, synaptic connectivity, and topographic innervation within target muscles, thus supporting proper motor function and neural circuit development [34]. Corroborating the validity, the GO terms related to “regulation of cell junction assembly”, “axon regeneration”, and “neuron projection regeneration” were also enriched (Fig. 6d).

Conversely, we conducted a pathway analysis of proteins negatively correlated with clinical alterations, indicating downregulation of these pathways as patients improve (Fig. 6d, Supplementary Fig. 6). Most significantly regulated pathways were associated with the activation of the complement system. Previously, complement cascade activation had been linked to degeneration of nerve cells [35].

Supporting that the intrathecal immune response is diminished in clinically improving SMA patients, several semantically-related GO terms such as “Humoral response mediated by circulating immunoglobulin”, “B cell receptor signaling pathway”, and “B cell activation”, which reflected the plethora of immunoglobulins that was negatively correlated with HFMSE alterations, were found (Fig. 6d). Together with the reduced CSF lymphocyte counts, these facts point towards a less activated immune response phenotype in nusinersen responders among SMA patients.

## Discussion

In this 3.5-year longitudinal study involving 24 patients with SMA type 2 or 3, a workflow of two complementary MS protein quantification methods to analyze CSF was employed. Treatment with nusinersen led to an overall increase in the HFMSE score but with significant inter-patient variability. We observed an inverse correlation between the abundance change of qAlb, glucose, and lactose, and the progression of the disease, suggesting their potential as monitoring biomarkers. Further exploration of protein profiles uncovered insights into pathways involved in “nervous system development” and “axogenesis”, as well as an association between clinical improvement and the upregulation of the interacting proteins DAG1 and B4GAT1, which have been previously identified as essential for neuromuscular integrity. Moreover, the study revealed a decrease in humoral factor proteins, such as complement factors and a negative correlation with clinical changes in immunoglobulin- and B cell-related pathways, as well as a reduction in cellular mediators like lymphocytes involved in the immune response in patients showing clinical improvement with nusinersen.

The role of pre-therapy parameters as predictors of nusinersen response remains unclear. In our study, treatment response was not influenced by readily identifiable baseline factors. Precisely, while previous studies in children yielded inconsistent results regarding the influence of *SMN2* copy number [36–38], our observations in adult patients suggest that *SMN2* copy number does not significantly affect treatment response. Furthermore neither baseline HFMSE score, SMA type, functional classification, age at treatment onset nor duration of symptoms reliably predicted therapy outcomes in our cohort. These findings underscore the complexity of nusinersen response and emphasize the necessity of identifying alternative biomarkers for predicting treatment effectiveness and therapy monitoring.

QAlb is a marker for blood–brain barrier impairment, as albumin in the CSF is exclusively derived from the bloodstream [39]. While the increase in qAlb may be due to repeated lumbar punctures [40], it could also be influenced by the nusinersen treatment itself. To differentiate this, a control group of untreated SMA patients would be needed, but such a design would be ethically unfeasible. Finally, the progressive nature of SMA may explain the increase, as elevated qAlb levels have been linked to neurodegenerative diseases [41]. Supporting this, negative correlation between qAlb and clinical improvement in our cohort suggests more significant increases at advanced disease stages.

As compared to targeted biomarker-based strategies relying on markers like the neuronal destruction marker neurofilament light chain (NfL), we followed an exploratory, non-hypothesis-driven approach aiming to provide a more detailed understanding of individual proteins and their associated molecular pathways. This enabled the identification of specific biomarkers that offer deeper insights into the mechanisms underlying nusinersen-induced CSF changes, and therapeutic response. The use of advanced fractionation techniques in conjunction with TMT labeling and high-resolution mass spectrometry provided superior protein coverage, revealing distinct pathways linked to clinical improvement. TMT, known for its heightened sensitivity in quantitative investigations and low incidence of missing values [42], particularly excels in the identification of noteworthy protein changes within the context of less abundant proteins. In addition, precision inherent in TMT-based protein quantification markedly strengthens the overall reliability of results [42, 43]. By combining this approach with the more accurate LFQ method, we capitalized on the strengths of both methodologies. This dual strategy, which revealed overlapping results and key insights, ensuring robustness and depth, represents a methodological advancement over previous studies, which often employed less sensitive or less comprehensive approaches.

In our longitudinal analysis, several overlapping DEPs including the elevated FCGBP and reduced GAPDH, GRIA4, and SEMA7A proteins were found. Noteworthy, these were regulated in the same direction in an independent study by Faravelli et al. [18], corroborating the validity of our approach. Overall, the 14 identified overlapping DEPs demonstrated high potential as biomarkers due to a high percentage of individual patients exhibiting the corresponding regulation for each protein, and should thus be validated in future studies with larger cohorts.

Among the shared proteins, we found the functionally validated interacting receptor–ligand pair OMG and RTN4R to be downregulated over time. Interestingly, the interaction between them has been described to be a pivotal impediment to successful axon regeneration in the adult central nervous system following injuries [31]. Thus, the observed downregulation of both proteins in response to nusinersen therapy in our study could suggest an augmented neuronal regenerative potential through this mechanism, paralleled by clinical improvement. In addition, both OMG and RTN4R were downregulated at all post-therapy timepoints in at least 80% of patients, with RTN4R being consistently downregulated in 100% of patients from month 10 onwards, except for one data point (at month 34). This makes RTN4R a potential longitudinal biomarker, though larger validation cohorts are necessary to confirm its utility.

In our quest for biomarkers to monitor therapy effectivity, we discovered a positive correlation between HFMSE increase and the elevation of the interacting proteins DAG1 and B4GAT1. DAG1, identified as α-dystroglycan, is a transmembrane protein crucial for linking the cytoskeleton to extracellular-matrix proteins and undergoes O-linked posttranslational modifications, essential for its role as an extracellular-matrix receptor in tissues like skeletal muscle and brain [33, 44, 45]. Hypoglycosylation of α-dystroglycan is implicated in various genetic muscular dystrophies [45, 46]. On the other hand, B4GAT1 is integral to the glycosylation pathway of the α-dystroglycan subunit [32, 47]. It acts as a priming enzyme for the xylosyl- and glucuronyltransferase (LARGE1), initiating the LARGE-dependent repeating disaccharide in the O-mannosylation protein glycosylation pathway [47]. In line with our findings of DAG1 and B4GAT1 positively correlating with an increase in the HFMSE score in response to nusinersen treatment, LARGE1 was recently identified as a disease marker for SMA, enabling differentiation between responders and non-responders to nusinersen therapy [21]. In addition, we identified DAG1 and B4GAT1 as potential monitoring biomarkers for the individual clinical course setting cutoffs. While DAG1 increases of ≥ 0.25 log2 intensity were linked to improvement or stability and decreases of ≥ 0.25 log2 intensity were associated with no improvement or worsening, B4GAT1 increases correlated with improvement or stability, and decreases > 0.5 log2 intensity were exclusive to patients with worsening or no improvement. However, validating these cutoffs for clinical application would require significantly larger cohorts. These results suggest that the mechanisms involving DAG1 and B4GAT1 may contribute to the observed motor skill alterations in response to nusinersen therapy.

We identified a strong negative correlation between the complement factors C7, C4A, and C1S across both proteomic datasets. In addition, the pathways “Complement activation, classical pathway” and “Complement activation” were among the top three most significantly negatively correlated with clinical changes in our cohort. In line with this, previous studies demonstrated that reduced complement activation leads to a more favorable recovery trajectory of motor and sensory nerve functions in SMA patients [48, 49]. In addition, upregulation of classical pathway proteins contributes to microglia-mediated synapse elimination within spinal sensorimotor circuits in SMA mouse models [48, 50]. The findings suggest that targeting the complement system alongside nusinersen may offer a novel approach to enhance treatment efficacy, as supported by evidence in preclinical models [50].

The reduced CSF lymphocyte counts and negatively correlated pathways such as “Humoral response mediated by circulating immunoglobulin”, “B cell receptor signalling pathway” and “B cell activation” in clinically improving SMA patients suggest a less activated immune phenotype in nusinersen responders. This is consistent with recent findings that pro-inflammatory pathways identified in the scRNA-seq data of CSF before treatment were absent post-treatment [51], indicating nusinersen’s potential regulatory effects on SMA-associated neuroinflammation. However, it remains unclear whether neuroinflammation in SMA is a response to neurodegeneration or contributes to its pathogenesis, though SMA is generally considered a non-cell autonomous disease, with inflammatory changes typically preceding motor neuron loss [52]. Further investigation to better understand the relation of the identified downregulated factors of the humoral response, i.e., immunoglobulins and complement factors, is warranted. It is of particular interest to shed light on the nature of the lymphocytes, which would help to find the causal link between their diminished numbers and the reduced humoral factors of the innate immunity in responding patients. This could open the avenue for more tailored therapies for SMA and combination treatments with nusinersen.

## Supplementary Information

Below is the link to the electronic supplementary material.

Supplementary file1 (PDF 2658 KB)

## Data Availability

MS proteomics data have been deposited to the ProteomeXchange Consortium (http://proteomecentral.proteomexchange.org) via the PRIDE partner repository with the dataset identifier PXD054900.

## Abbreviations

AGC: Automatic gain control
ASO: Antisense oligonucleotide
B4GAT1: Beta-1,4-glucuronyltransferase 1
CSF: Cerebrospinal fluid
DAG1: α-Dystroglycan
DDA: Data-dependent acquisition
DEP: Differentially expressed protein
FA: Formic acid
FDR: False discovery rate
GO: Gene Ontology
HFMSE: Hammersmith Functional Motor Scale-Expanded
LARGE1: Xylosyl- and glucuronyltransferase
LFQ: Label-free quantification
MeCN: Acetonitrile
MS: Mass spectrometry
OMG: Oligodendrocyte myelin glycoprotein
qAlb: Albumin quotient (CSF/serum)
RTN4R: Reticulon-4 receptor
SMA: Spinal muscular atrophy
SMN: Survival motor neuron
TFA: Trifluoroacetic acid
TMT: Tandem mass tag
Human genes: SMN1, SMN2
