# Supplementary material for: Long-term CSF responses in adult patients with spinal muscular atrophy type 2 or 3 on treatment with nusinersen
Source: J Neurol. 2025 Mar 14;272(4):270. doi: 10.1007/s00415-025-12984-7 (PMC11909034; doi:10.1007/s00415-025-12984-7)
Supplement: Supplementary file 1 — Supplementary file1 (PDF 2658 KB) [file 415_2025_12984_MOESM1_ESM.pdf]

Long-term CSF responses in adult patients with spinal muscular atrophy type 2 or 3 on treatment with nusinersen

Jounal of Neurology

Supplementary tables and figures

Gina Cebulla[1,2], Ling Hai[1,2], Uwe Warnken[1], Cansu Guengoer[3], Dirk C. Hoffmann[1,2], Mirjam Korporal-Kuhnke[3], Brigitte Wildemann[3], Wolfgang Wick[1,2], Tobias Kessler[1,2,*], and Markus Weiler[3,*]

[1]Clinical Cooperation Unit Neurooncology, German Cancer Consortium (DKTK), German Cancer Research Center (DKFZ), Heidelberg, Germany
[2]Neurology and Neurooncology Program, National Center for Tumor Diseases, Heidelberg University Hospital, Heidelberg, Germany
[3]Department of Neurology, Heidelberg University Hospital, Heidelberg, Germany

*Equal contribution

Corresponding authors:
Markus Weiler, MD
Department of Neurology
Heidelberg University Hospital
Im Neuenheimer Feld 400
D-69120 Heidelberg
Germany
Phone: +49 6221 567504
Fax: +49 6221 565461
Email: markus.weiler@med.uni-heidelberg.de

or

Tobias Kessler, MD
Department of Neurology
Heidelberg University Hospital
Im Neuenheimer Feld 400
D-69120 Heidelberg
Germany
Phone: +49 6221 567504
Fax: +49 6221 565461
Email: tobias.kessler@med.uni-heidelberg.de

Supplementary table 1 Individual patient characteristics

**Patient No.**	**SMA type**	**SMA class**	**Age [years]**	**Gender**	**Duration of symptoms [years]**	***SMN1* Genetics**	***SMN2* [n]**	**Wheelchair**	**HFMSE baseline**	**ΔHFMSE after 10 months**
SMA01	3b	W	18	m	6	Het. Δ7/8, c.283G < C	2	yes	44	3
SMA02	3a	S	24	f	20	Het. Δ7/8, c*3 + 6T > G	2	yes	36	2
SMA03	3b	S	27	m	19	Homozygous Δ7/8	4	yes	26	9
SMA04	3b	W	38	m	23	Homozygous Δ7/8	3	no	41	7
SMA05	3a	S	33	f	23	Homozygous Δ7/8	2	yes	10	0
SMA06	3a	W	48	f	46	Homozygous Δ7/8	4	no	15	12
SMA07	3b	W	50	m	34	Homozygous Δ7/8	4	no	56	0
SMA08	3b	S	50	m	46	Homozygous Δ7/8	4	yes	7	0
SMA09	3b	S	46	f	37	Het. Δ7/8, c.821C > T	3	yes	6	0
SMA10	2	N	29	f	29	Homozygous Δ7/8	4	yes	0	0
SMA11	3b	W	35	m	21	Homozygous Δ7/8	3	no	49	1
SMA12	2	S	18	f	17	Homozygous Δ7/8	3	yes	2	4
SMA13	3b	W	34	m	23	Homozygous Δ7/8	4	no	57	5
SMA14	3a	S	20	f	17	Homozygous Δ7/8	3	yes	16	0
SMA15	3b	S	27	m	14	Homozygous Δ7/8	4	yes	25	0
SMA16	3a	S	31	m	29	Homozygous Δ7/8	3	yes	10	2
SMA17	3a	S	41	m	39	Homozygous Δ7/8	4	yes	9	-3
SMA18	3a	S	30	f	30	Homozygous Δ7/8	3	yes	2	0
SMA19	3a	S	33	f	32	Het. Δ7/8, c.90_91insT	2	yes	29	3
SMA20	2	N	22	m	21	Homozygous Δ7/8	3	yes	0	0
SMA21	3b	W	57	m	39	Homozygous Δ7/8	4	no	59	1
SMA22	3a	S	21	m	20	Homozygous Δ7/8	4	yes	19	2
SMA23	3a	S	23	f	19	Homozygous Δ7/8	3	yes	25	3
SMA24	3b	S	40	f	29	Homozygous Δ7/8	3	yes	8	1
**Mean**	**N/A**	**N/A**	**33.1**	**N/A**	**26.4**	**N/A**	**3**	**N/A**	**23**	**2.2**
**SEM**	**N/A**	**N/A**	**2.3**	**N/A**	**2.1**	**N/A**	**0.2**	**N/A**	**3.9**	**0.7**

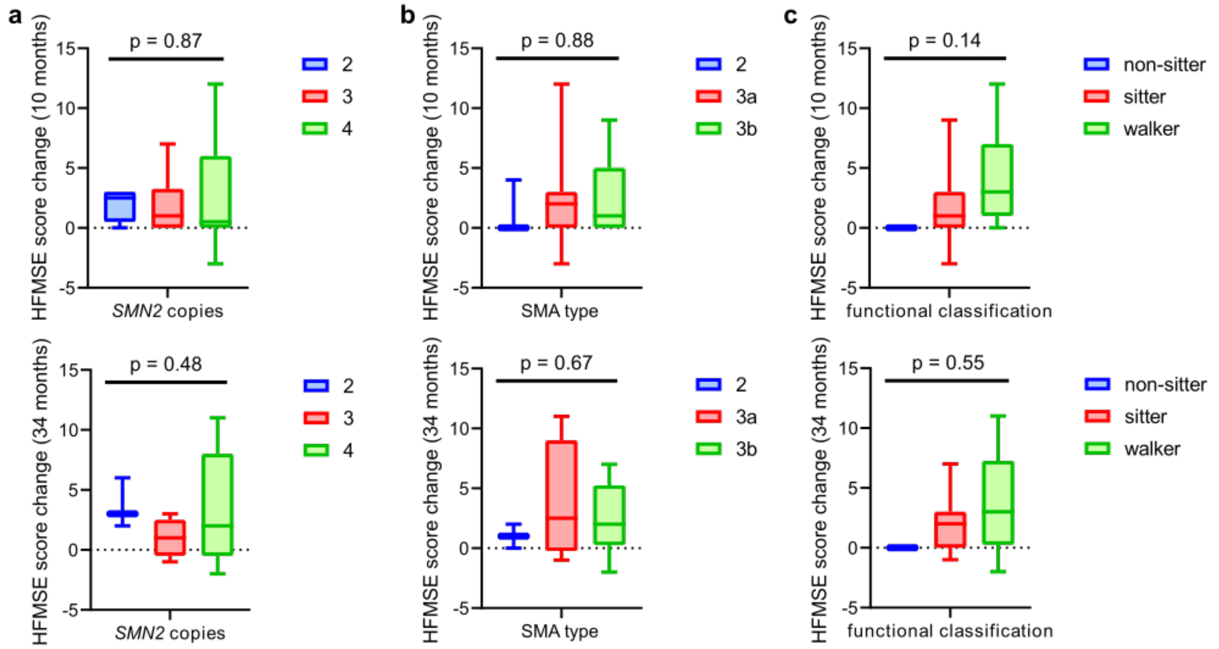

Supplementary Fig. 1 HFMSE score change by subcategory. **A**, Based on *SMN2* copy numbers. **B**, Based on SMA type. **C**, Based on functional classification. **Upper row**, Clinical response measured after 10 months of treatment with nusinersen. **Lower row**, Clinical response measured after 34 months of treatment with nusinersen.

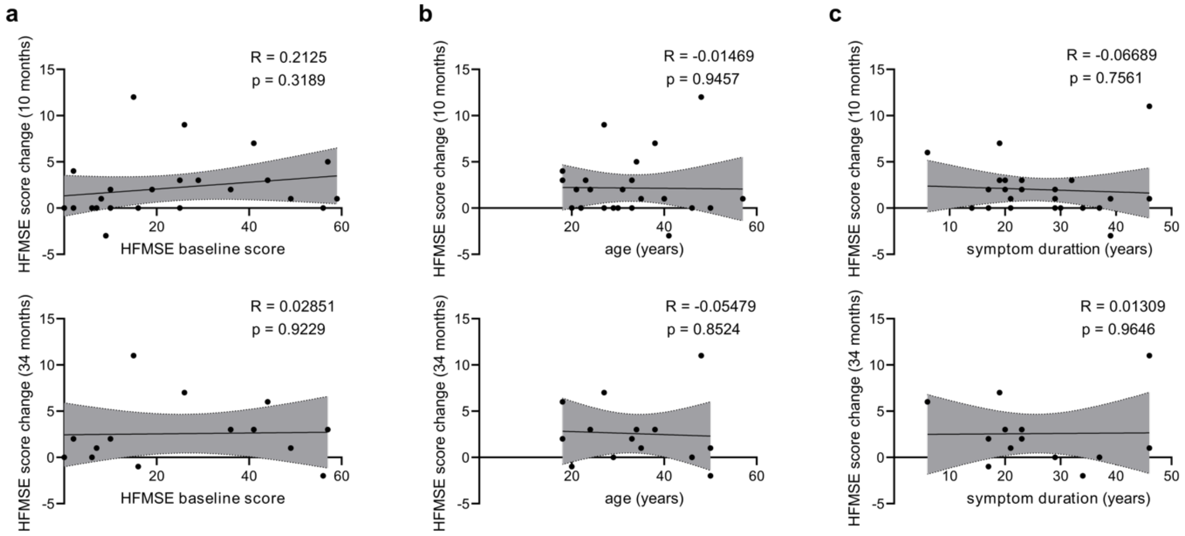

Supplementary Fig. 2 Correlation analysis of clinical response with patient characteristics in our study cohort. **A**, Correlation with HFMSE baseline score. **B**, Correlation with age in years. **C**, Correlation with symptom duration. **Upper row**, Clinical response measured after 10 months of treatment with nusinersen. **Lower row**, Clinical response measured after 34 months of treatment with nusinersen.

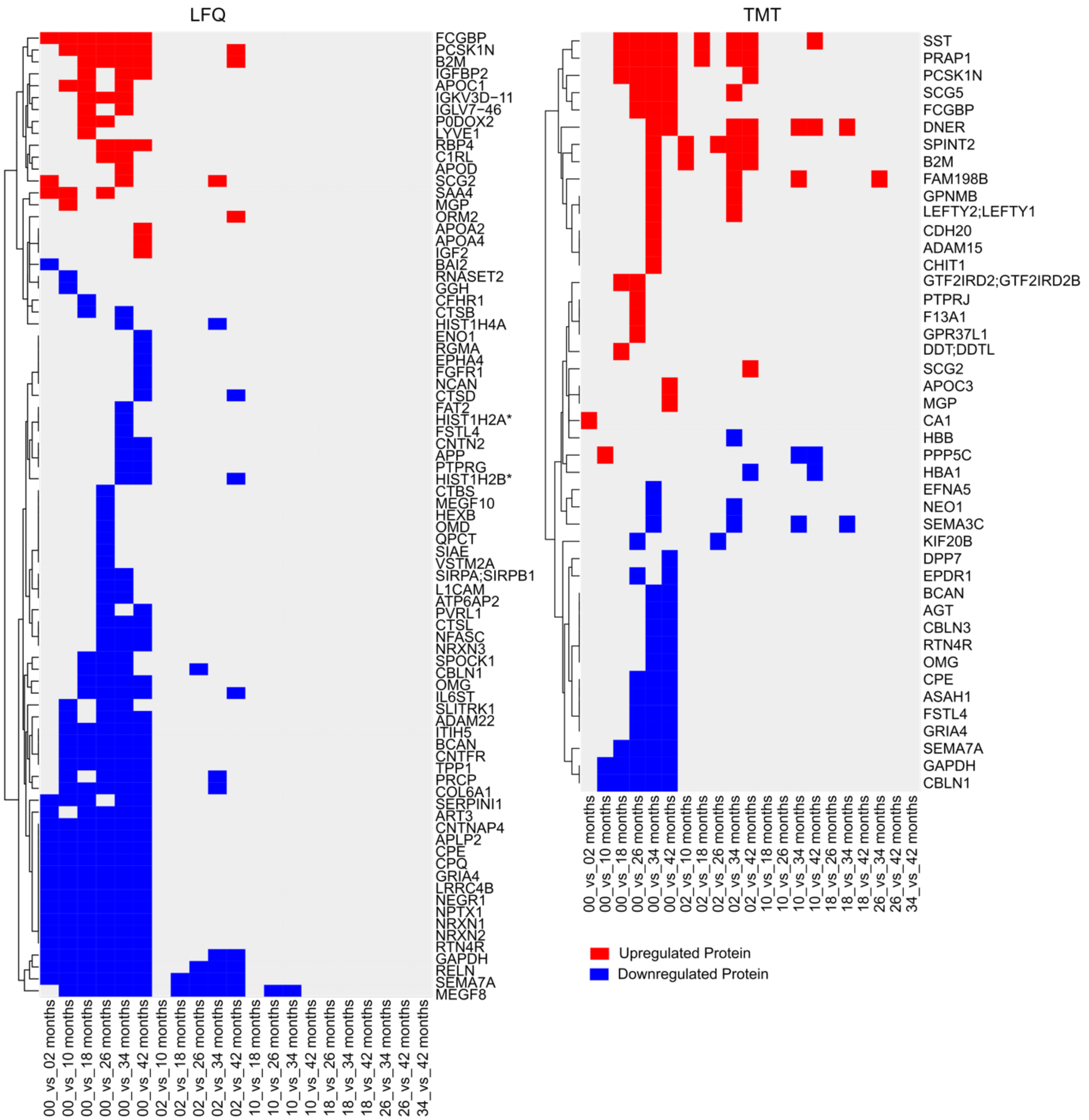

Supplementary Fig. 3 Differentially expressed protein (DEP) analysis for each timepoint. **a** DEPs in the LFQ analysis. **b** DEPs in the TMT analysis.

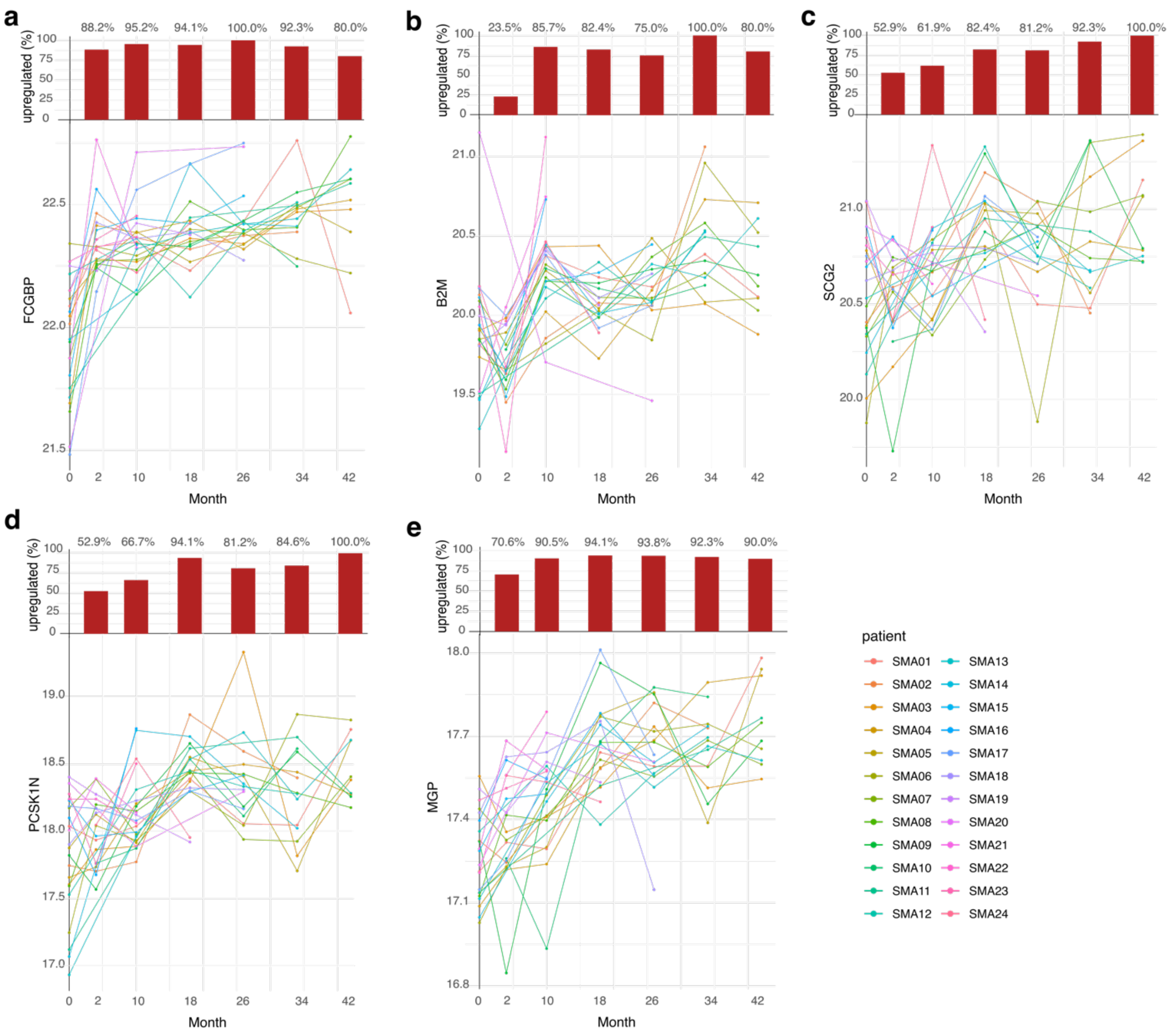

Supplementary Fig. 4 Temporal dynamics of upregulated longitudinal DEPs in individual patients and the proportion of patients at each time point with upregulated levels of the respective protein compared to baseline. The selected proteins shown are **a** FCGBP, **b** B2M, **c** SCG2, **d** PCSK1N, and **e** MGP.

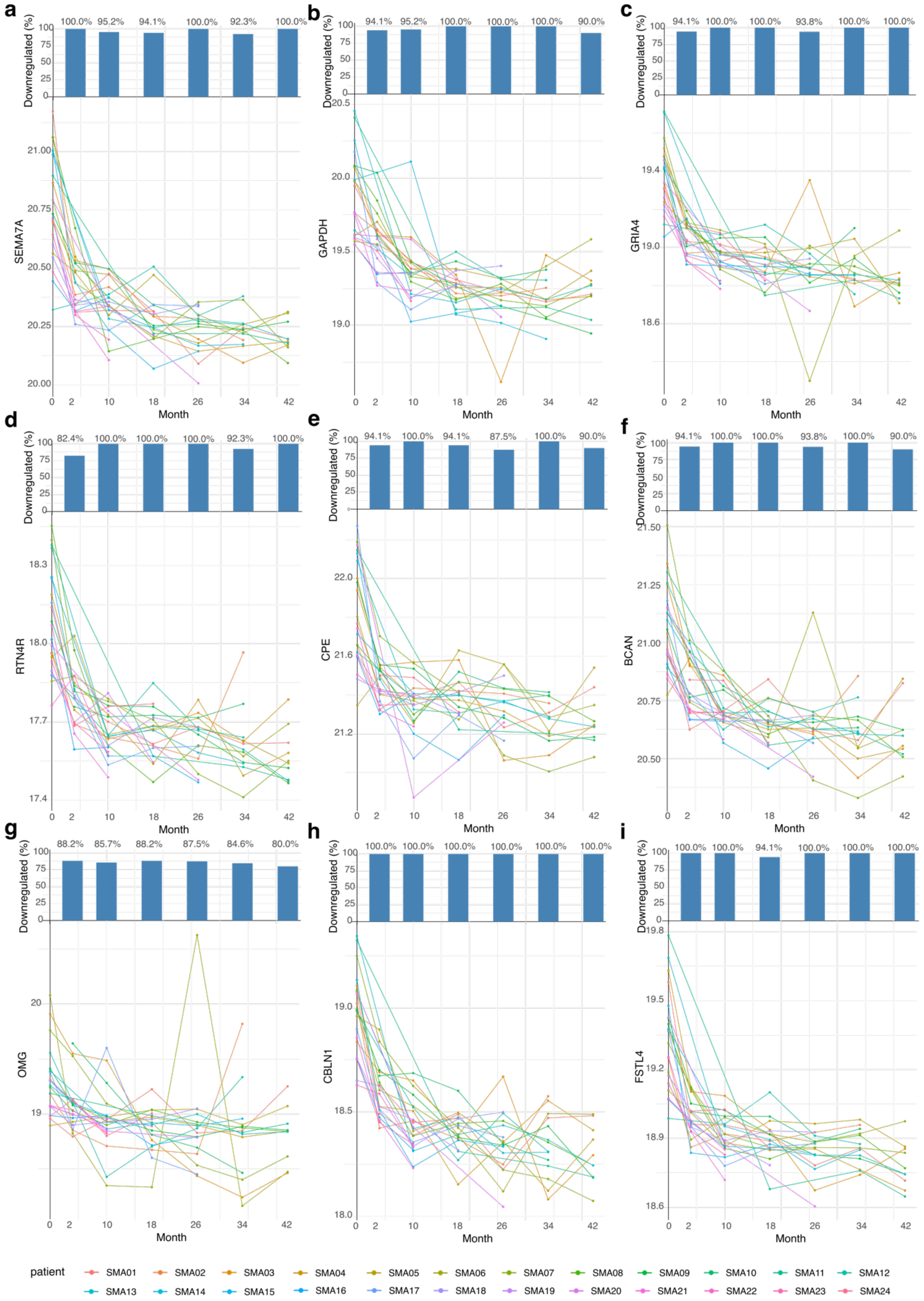
a
100.0% 95.2% 94.1% 100.0% 92.3% 100.0%
Downregulated (%)
SEMA7A
b
94.1% 95.2% 100.0% 100.0% 100.0% 90.0%
GAPDH
c
94.1% 100.0% 100.0% 93.8% 100.0% 100.0%
GRIA4
d
82.4% 100.0% 100.0% 100.0% 92.3% 100.0%
RTN4R
e
94.1% 100.0% 94.1% 87.5% 100.0% 90.0%
CPE
f
94.1% 100.0% 100.0% 93.8% 100.0% 90.0%
BCAN
g
88.2% 85.7% 88.2% 87.5% 84.6% 80.0%
OMG
h
100.0% 100.0% 100.0% 100.0% 100.0% 100.0%
CBLN1
i
100.0% 100.0% 94.1% 100.0% 100.0% 100.0%
FSTL4
Month
patient
SMA01 SMA02 SMA03 SMA04 SMA05 SMA06 SMA07 SMA08 SMA09 SMA10 SMA11 SMA12
SMA13 SMA14 SMA15 SMA16 SMA17 SMA18 SMA19 SMA20 SMA21 SMA22 SMA23 SMA24

Supplementary Fig. 5 Temporal dynamics of downregulated longitudinal DEPs in individual patients and the proportion of patients at each time point with downregulated levels of the respective protein compared to baseline. The selected proteins shown are **a** SEMA7A, **b** GAPDH, **c** GRIA4, **d** RTN4R, **e** CPE, **f** BCAN, **g** OMG, **h** CBLN1 and **i** FSTL4.

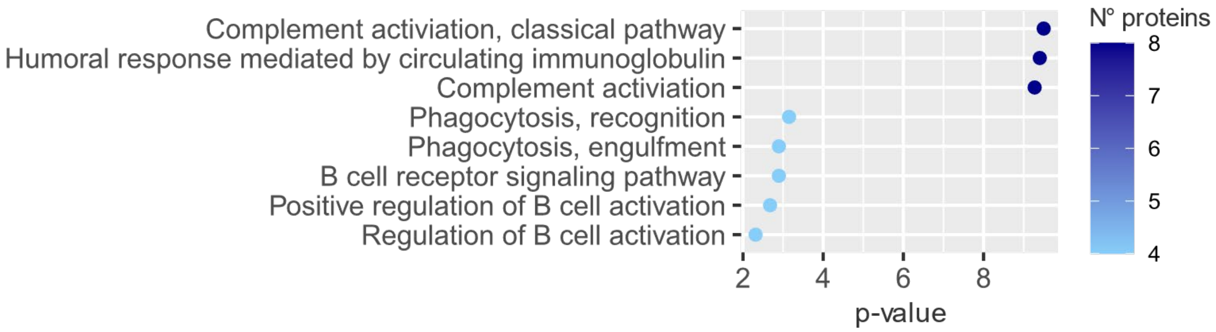

Supplementary Fig. 6 GO term analysis of negatively correlated protein changes with HFMSE alterations in LFQ proteomics. There are no GO terms of negatively correlated protein changes with HFMSE alterations in LFQ proteomics.
